# Micropropagation and somaclonal variation in Iranian genotypes of garlic (*Allium sativum* L.)

**DOI:** 10.1371/journal.pone.0331782

**Published:** 2025-09-10

**Authors:** Mohammad-Hossein Afzaz, Javad Mozafari, Sepideh Sanjari

**Affiliations:** 1 Genetic Research Department, Seed and Plant Improvement Institute, Agricultural Research, Education and Extension Organization, Karaj, Iran; 2 Agricultural Research, Education and Extension Organization, Tehran, Iran; 3 Agronomy and Plant Breeding Department, College of Aburaihan, University of Tehran, Tehran, Iran; Ataturk University: Ataturk Universitesi, TÜRKIYE

## Abstract

Garlic is an important bulb vegetable which is used for both culinary and medical purposes worldwide. *In vitro* propagation is considered a promising technic for production and conservation of disease-free garlic seed. The efficiency of *in vitro* culture was studied for micropropagation of native Iranian garlic genotypes. A factorial experiment based on a completely randomized design with three replications was conducted to optimize the *in vitro* culture media components for establishment, regeneration and conservation of four Iranian garlic genotypes. The highest number of bulblets were obtained and established on MS medium supplemented with 1.5 mgL^-1^ BA (benzyl adenine) + 0.5 mgL^-1^ IBA (indole-3-butyric acid) and 0.5 mgL^-1^ 2-iP (2-isopentenyl adenine) + 0.25 mgL^-1^ NAA (naphthalene acetic acid), respectively. In the regeneration phase, however, the highest number of bulblets regenerated from *in vitro* grown plants on MS medium supplemented with 0.5 mgL^-1^ 2-iP + 0.1 mgL^-1^ NAA. A drastic increase in bulblet formation was also observed on this culture medium during conservation phase. At least 20 bulblets were formed per each explant in all genotypes in the first subculture. Interestingly, the bulblet formation in the second subculture was 4–5 times more than the first subculture, indicating a very efficient regeneration rate in micropropagation of Iranian garlic genotypes. Moreover, RAPD (Randomly Amplified Polymorphic DNA) markers were used to evaluate the genetic stability in regenerated garlic plantlets. All fragments amplified by five RAPDs were the same in regenerated plantlets and their mother plants showing no somaclonal variation in micropropagated Iranian garlics. Our results indicated that *in vitro* protocols used in this study can provide an efficient system for regeneration and conservation of garlic germplasm in an *in vitro* gene-bank.

## Introduction

Garlic (*Allium sativum* L.) is a monocotyledonous plant belonging to the Alliaceae (onion) family and is considered the second most extensively distributed species of Allium genus after onion in the world [1]. Garlic is a diploid species (2n = 2x = 16) with a large genome size (15.9 gigabase pairs) which is grown from temperate to subtropical climates worldwide. Its center of origin and primary center of diversity is located in Northern Iran and Central Asia [2]. The plant is used as a spice and vegetable or for medicinal purposes across the globe. It is considered to be useful for boosting the immune system and preventing cancer due to varied bioactive compounds and micronutrients in its bulbs [3,4]. Such facts have caused a significant expansion of garlic plantation so garlic annual production has increased from 5.78 to 28.05 million tons in the last three decades [5,6]. The main garlic producers in the world are China, India and Bangladesh [7]. Garlic is an important native plant in Iran with 58278 tons of production annually. Garlic is usually known as a sterile plant [8,9], because infertile varieties of garlic (non-bolting) reproduce by vegetative (clonal) propagation and some fertile varieties (bolting) produce flower but seeds are usually unfertile [8,10,11]. So, the main cultivation method of garlic is asexual reproduction through planting the cloves of bulbs [12]. Vegetative propagation strongly limits genetic variability that is used for breeding economically important traits such as environmental tolerance. It also leads to impracticality of major breeding strategies in garlic [13,14]. Moreover, garlic as vegetatively propagated plant is susceptible to diseases particularly viral infections which leads to reduced quality and quantity of crop yield. In addition, viruses are easily transmitted from one generation to the next through bulbs [15]. This propagation method leads to the transmission of viral infections caused by viruses such as Potyviruses, Carlaviruses, and Allexiviruses, resulting in a significant decrease in crop yield [16,17]. Therefore, garlic propagation using cloves has several disadvantages including low rate of multiplication, high cost and short term of maintenance, pathogens transmission via generations which could lead to significant crop loss [1,13].

Due to such problems, conventional garlic propagation cannot also be considered as an efficient system for conservation of garlic genetic resources or propagation and distribution of seed garlic clones. On the other hand, regional and global interdependency for genetic resources of important food crops such as garlic is growing due to increasing climate change challenges. Under these circumstances development of an efficient system for production, propagation and movement of disease-free garlic germplasm across the world is a pressing necessity. Developing *in vitro* conservation and micropropagation protocols is a prerequisite for achieving such an efficient system in vegetatively propagated crops as it has been emphasized in several international agreements [9]. Unfortunately, no reliable and efficient *in vitro* protocol has ever been reported for conservation and micropropagation of garlic germplasm. Biotechnological tools including organogenesis, somatic embryogenesis, and meristem culture have shown potentials in micropropagation of garlic and production of *in vitro* garlic plants in recent years [13,18]. Previous reports illustrated different *in vitro* culture methods such as callus, root tip, meristem, shoot apex and stem-discs employed to micropropagate various garlic cultivars [19–22]. Also progress has been made in using meristem culture to regenerate virus-free plantlets as a basis to produce virus free nucleus seed stock in several vegetatively propagated crops [18,23].

Theoretically, regenerated plants in tissue culture should be genetically uniform and stable. However, tissue culture may generate unexpected variation known as somaclonal variation which could be the main problem of *in vitro* grown plants [24]. The rate of such variation may be affected by factors including propagation methods, culture medium, environmental condition, explant source, number and duration of subcultures [25,26]. Morphological, physiological, biochemical and molecular markers are generally used to study somaclonal variation. Molecular markers based on DNA such as AFLP (amplified-fragment length polymorphism), RAPD (random-amplified polymorphic DNA) and SSR (simple-sequence repeats) among them are not affected by the environment and allow the detection of variations at early stages [18,26–29]. RAPD markers have been successfully used for determining genetic diversity and somaclonal variation in garlic [30,31].

Availability of a high frequency *in vitro* multiplication as well as virus eradication and meristem culture techniques can provide a viable strategy for *in vitro* conservation, micropropagation and production safe distribution of garlic clones. So, the aim of current study was to find an optimized *in vitro* culture protocol for micropropagation and conservation of widely grown Iranian native garlic genotypes.

## Materials and methods

### Plant material

Bulbs of four native Iranian garlic (*A. sativum* L.) genotypes which are widely grown in main garlic producing regions of the country including Isfahan1, Isfahan2, South Khorasan1 and South Khorasan2 were obtained from the National Gene-Bank of Iran. Garlic cloves of each genotype were separated from their bulbs and cold treated at 4°C for two weeks. After peeling the outer protective layer, cloves were pre-sterilized with distilled water 4–5 times and then surface-sterilized with 70% ethanol for 60 seconds, followed by 20% sodium hypochlorite solution (NaOCl) for 25 minutes under laminar airflow. Sterilized cloves were immersed and rinsed in sterile distilled water three times with different immersing time period (3,3, 5 minutes) to remove excess NaOCl. Cloves of each genotype were cultured in sterile glass test tubes containing MS medium [32] solidified with 3% agar and were incubated in the growth chamber under 20°C and 16/8h light and dark regime, until cloves sprouted. After sprouting the storage leaves of the cloves were removed and shoot meristems from basal portion of cloves were divided into 4–6 sections (2–3 mm in diameter) and used as explants.

### *In vitro* shoot tip culture, regeneration and conservation

To find an optimized culture media *in vitro* shoot tip culture of four Iranian garlic genotypes was conducted on MS media supplemented with four combinations of plant growth regulators (Table 1) in a factorial experiment based on a completely randomized design with three replications. It is noteworthy that solid MS medium fortified with 3% (w/v) sucrose was used as basal media for *in vitro* establishment and regeneration of garlic. The cultures were incubated in the growth chamber under 20 ± 2 °C and 16 h light/ 8 h dark regime with 1500 lux of light intensity.

**Table 1:** Garlic genotypes and PGR combinations studied in a factorial experiment based on completely randomized design to optimize culture medium for establishing *in vitro* garlic culture.

Factor 1 (Genotypes)	Factor 2 (Combinations of PGRs (mg L^-1^))
Isfahan1	0.5 2-iP + 0.25 NAA
Isfahan2	0.5 2-iP + 0.5 IBA
South Khorasan1	1 BA + 1 NAA
South Khorasan2	1.5BA + 0.5 IBA

NAA, naphthalene acetic acid; IBA, indole-3-butyric acid; BA, benzyl adenine; 2-iP, 2- isopentenyl adenine.

The regeneration frequency and characteristics of *in vitro* grown plantlets were then examined in two independent factorial experiments in a completely randomized design with three replications. In the first experiment Effects of different concentrations of plant growth regulators (PGRs); NAA (0, 0.1 and 0.2 mgL^-1^) and 2-iP (0, 0.5 and 1 mgL^-1^) was studied on the regeneration of garlic genotype Isfahan 1. In the second experiment, effects of the optimized fixed-level of NAA (0.1 mgL^-1^) along with different levels of 2-iP (0.5, 1, 2 and 4 mgL^-1^) were assessed on micropropagation of the four garlic genotypes.

For assessing the *in vitro* conservation capacity of Iranian garlic genotypes, shoot meristem of *in vitro* plantlets were divided into four pieces. Each piece was grown on a culture medium for 4 weeks. After four weeks, the number of bulblets per culture were recorded. Then, the second subculture was done and the number of bulblets per culture were measured again after four weeks. This experiment was conducted as a factorial experiment on a completely randomized design with three replications in which the effects of sub-culturing and genotypes on frequency of regeneration were studied.

### Assessment of somaclonal variation of *in vitro* seedlings

*In vitro* grown plantlets were first acclimatized to natural light, by transferring culture tubes to rooms with natural light flow for 3 days before being moved to the greenhouse. Then, plantlets were transferred from the culture tubes to the pots with oven sterilized sand. For doing that the plantlets were taken out gently on a disinfected work surface using sterilized forceps. The plantlets were then washed gently with sterilized distilled water to remove culture medium. After transferring the seedlings to the pots and providing them with adequate water, a transparent cover was placed over each pot to minimize excessive water evaporation. The bottom of each pot was also placed in a tray containing water to ensure a continuous supply of moisture. During the first week, the covers were removed for 15 minutes daily. Towards the end of the first week, this process was performed twice a day. At the beginning of the second week this was increased to 30 minutes, gradually increasing to 1–2 hours twice a day by the middle of the second week. Finally, three days before the covers were permanently removed, they were lifted for four hours daily to allow for the gradual adjustment of stomata and cuticle formation. By the end of the fourth week, the plants transferred to the greenhouse were replanted into pots containing a 1:1 mixture of soil and sand. Morphological traits were then evaluated over six months according to the qualitative descriptive characteristics outlined in the IPGRI international descriptor [33] for the *Allium* genus.

### DNA isolation and RAPD analysis

Genomic DNA of young shoots of *in vitro* plantlets of all genotypes and their mother plants were extracted according to the Doyle method [34]. Briefly, leaves samples (0.8 g) of genotypes were ground with liquid nitrogen. Then, extraction buffer (20 mM EDTA, 1.4 M NaCl, 100 mM Tris-HCl, 2% CTAB, PVP and Mercaptoetanol) and 800 μl Mercaptoetanol were added, vortexed for 10s and incubated at 65^0^C for an hour. Then, 450 μl of each chloroform and isoamylalcohol were added to the samples and inverted well and then centrifuged at 13000 rmp for 15 min. Supernatants were collected and 800 μl ice-cold isopropanol were added to them and gently inverted and then centrifuged at 13000 rmp for 15 min. After discarding the supernatants, the pellets were dried and dissolved in 50 μl of distilled water. DNA quality and quantity was checked by 1% agarose gel electrophoresis and spectrophotometer, respectively.

Five common RAPD primers (Table 2) were used to assess the somaclonal variation between *in vitro* garlic plantlets and their parental clone. RAPD PCR reactions were done in a final volume of 25 µl according to Williams et al [35] with some changes. Each PCR reaction contained 1 μl of 500-ng DNA, 0.5 μl of primer (10 μM), 1 μl of 10 mM dNTPs, 2.5 μl of 10x PCR buffer, 0.5 μl of 5 U/μl Taq polymerase and 19.5 μl of sterile distilled water. The amplification profiles were consisted of an initial denaturing step for 3 min at 94°C; followed by 40 cycles at 94°C for 1 min, 33–42.5°C (depending on each primer’s annealing temperature) for 1 min, 72°C for 2 min and a final elongation step for 7 min at 72°C. The PCR products of each reaction were separated on 1.5% agarose gel electrophoresis containing 1x TBE buffer and also 1 Kb DNA ladder was electrophoresed beside the PCR products. After agarose gel staining with ethidium bromide, the amplified fragments were visualized under UV light using gel document instrument.

**Table 2:** Characteristics of RAPD Primers used to assess somaclonal variation in *in vitro* grown garlic plants.

Primer code	Sequence (5’-3’)	G + C content (%)	Annealing temperature
OPA-02	TGCCGAGCTG	70	36
OPD-01	ACCGCGAAGG	70	33
OPJ-12	GTCCCGTGGT	70	42.5
K-15	CTCCTGCCAA	60	34
K-20	GTGTCGCGAG	70	34

### Statistical analysis

Number of bulblets recorded for each culture in all three replications in the three experiments: *in vitro* culture establishment, regeneration and conservation of garlic. The collected data were analyzed as a factorial experiment on a completely randomized design using standard error (SE) according to the Snedecor and Cochran method [36] using statistical software SPSS.

## Result and discussion

Availability of a technically reliable and economically viable *in vitro* micropropagation system is of paramount importance for large scale germplasm conservation and distribution as well as seed garlic production. Most of the studies reported till now have focused on the preliminary steps of micropropagating *in vivo* garlic explants under *in vitro* conditions. In the study that we report here this issue has been taken to a level beyond the micropropagation of garlic clones. This study aimed at obtaining an optimized system for high frequency micropropagation of *in vitro* grown plantlets, in addition to just establishing *in vitro* culture. Number of experiments were conducted to examine and optimize important components of the system at both *in vitro* culture of *in vivo* garlic bulbs and *in vitro* subculture of bulblets or plantlets.

### Effects of PGRs on *in vitro* garlic growth of garlic shoot tips

**Fig 1:**
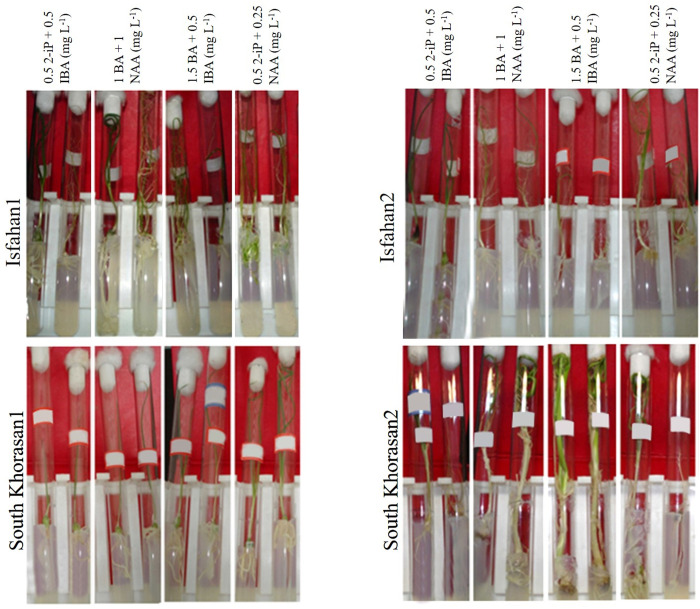
Bulblets formation of Iranian garlic genotypes on MS media containing different concentration of plant growth regulators.

**Fig 2:**
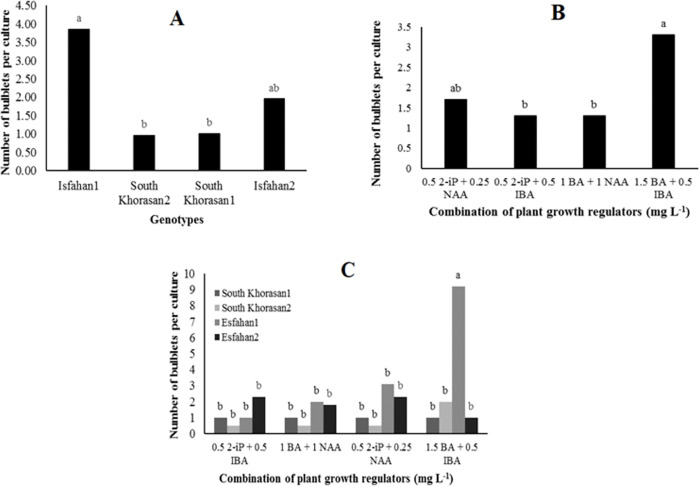
Mean number of bulblets per culture tube at establishment phase in four Iranian garlic genotypes. **(A)** Effects of genotype on number of bulblets, **(B)** Effect of plant growth regulator supplements on number of bulblets, **(C)** Effects of interaction of genotype and plant growth regulator supplement on number of bulblets. Different letters on bars indicate significant differences (P **<* *0.01 or 0.05).

Auxins and cytokinins among PGRs play vital roles in regenerating most of the plant species *in vitro* [37]. Analyzing number of bulblets formed in the fourth week of the primary culture (Fig 1) showed that the effects of genotype, PGR and their interaction were significant at <0.05 or 0.01 probability levels (Table 3). The highest number of bulblets (3.85/ explant) at this step was observed in Isfahan1 genotype (Fig 2A). Also, the culture media containing 1.5 mgL^-1^ BA + 0.5 mgL^-1^ IBA, and 0.5 mgL^-1^ 2-iP + 0.25 mgL^-1^ NAA, both produced higher number of bulblets (3.3 and 1.7/explant, respectively) while these numbers are not significantly different. (Fig 2B). Moreover, Isfahan1 genotype had the highest rate of bulblets formation (9.2/ explant) in those media mentioned above at *in vitro* culture establishment phase (Fig 2C). Genotypes responded differently to culture media which is in agreement with observation reported earlier by Izquierdo-Oviedo et al [18] and Metwally et al [38]. Roksana et al [39] also showed that semi-solid MS medium containing 0.5 mgL^-1^ 2-iP + 0.25 mgL^-1^ NAA induced bulblet formation in garlic. It seems that using the high level of cytokinin and low concentration of auxin was more effective for bulblet formation compared to same concentration of both PGRs in the establishment phase. This result is in contrast with results of Izquierdo-Oviedo et al [18] who found that similar or very close concentrations of auxins and cytokinins are more effective for establishing garlic *in vitro*.

**Table 3:** Analysis of variance regarding the effects of genotypes and plant growth regulators on bulblet formation at establishment phase of *in vitro* garlic culture.

Source of variation	df	Sum of squares	Mean square	F
Genotype (G)	3	92.172	30.724	6.606^**^
Growth regulators (PGRs)	3	44.297	14.766	3.175^*^
G * PGRs	9	132.891	14.766	3.175^**^
Experimental error	32	223.250	4.51	
Total	47	492.609		

** and * indicates significance at (P≤0.01) and (P≤0.05), respectively.

**Fig 3:**
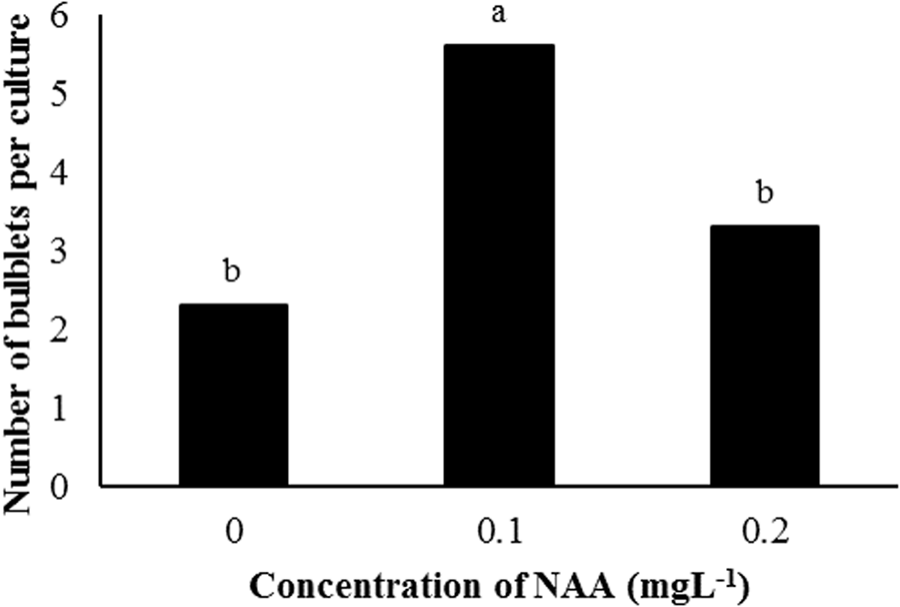
Effect of different concentration of NAA on number of bulblets per culture tube at regeneration phase in Isfahan1 genotype.

**Fig 4:**
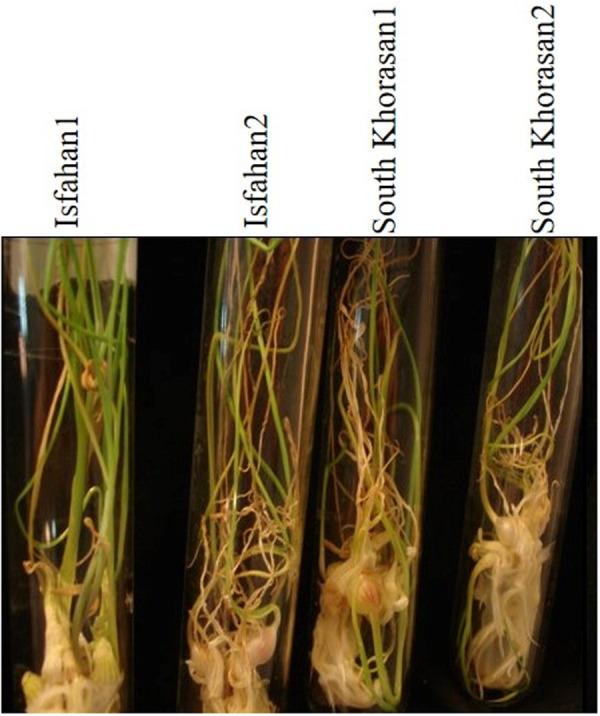
Bulblets formation at regeneration phase in four Iranian garlic genotypes on MS media culture containing 0.1 mgL^-1^ NAA and 0.5 mgL^-1^ 2-iP.

Previous studies illustrated that the presence of NAA and 2-iP in culture media promotes bulblet and shoot regeneration [40], while high doses of those PGRs can cause genetic variation *in vitro* [41]. So, culture media containing 2-iP and NAA were chosen to optimize the media for subculturing phase and regeneration of *in vitro* grown garlic bulblets/plantlets. Analysis of variance caused by different concentrations of 2-iP and NAA on Isfahan1 genotype showed that the effect of NAA was significant (p < 0.05 probability) on the number of bulblets per culture tube (Table 4). Moreover, bulblet formation was higher (6/ explant) in the culture media supplemented with 0.2 mgL^-1^ of NAA (Fig 3). In addition, the effects of 2-iP levels, genotypes and their interaction on mean number of bulblets formation at the subculture media or regeneration phase were not significant (Table 5). This indicated that, the culture medium containing 0.1 mgL^-1^ NAA and 0.5 mgL^-1^ 2-iP was the most suitable medium for garlic regeneration (Fig 4). Earlier studies have reported comparatively lower regeneration rates using the culture media supplemented with much higher PGR concentrations [10,42] such as 0.3 mgL^-1^ NAA and 3 mgL^-1^ 2-iP. This, is indeed a significant improvement which is reported by this research work. The finding of this study was also in line with results of Bhojwani [43] who found that number of shoots per culture was markedly increased on B-5 basal medium containing 0.1 and 0.5 mgL^-1^ of NAA and 2-iP, respectively. Dixit et al. [40] obtained the best medium for garlic shoot regeneration on the B-5 medium containing much higher levels of PGRs; 2.45 mgL^-1^ 2-iP and 0.537 mgL^-1^ NAA. Despite that, it seems basal (MS or B-5) medium supplemented with approximately five fold of 2-iP level to the NAA, similar to the ratio that we reported here.

**Table 4:** Analysis of variance regarding the effects of 2-iP and NAA on bulblets formation in Isfahan1 genotype at regeneration phase of *in vitro* garlic bulblets.

Source of variation	df	Sum of squares	Mean square	F
2-iP	2	115.630	57.815	3.122 ^n.s^
NAA	2	151.407	75.704	4.088 ^*^
2-iP*NAA	4	130.815	32.704	1.766 ^n.s^
Experimental error	18	333.333	18.519	
Total	26	731.185		

* indicates significance at (P ≤ 0.05) and ^n.s^ means no statistical difference.

**Table 5:** Analysis of variance regarding the effects of 2-iP and genotype on bulblets formation on the regeneration of *in vitro* bulblets in garlic.

Source of variation	df	Sum of squares	Mean square	F
Genotype (G)	3	48.396	16.132	1.558 ^n.s^
2-iP	3	15.229	5.076	0.49 ^n.s^
G * 2-iP	9	102.521	11.391	1.1 ^n.s^
Experimental error	32	331.333	10.354	
Total	47	497.479		

n.s means no statistical difference.

**Fig 5:**
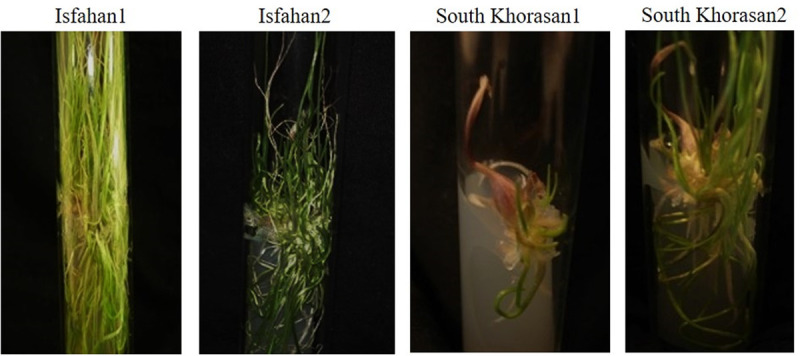
Bulblets formation at second subculturing in four Iranian garlic genotypes on MS media containing 0.1 mgL^-1^ NAA and 0.5 mgL^-1^ 2-iP.

**Fig 6:**
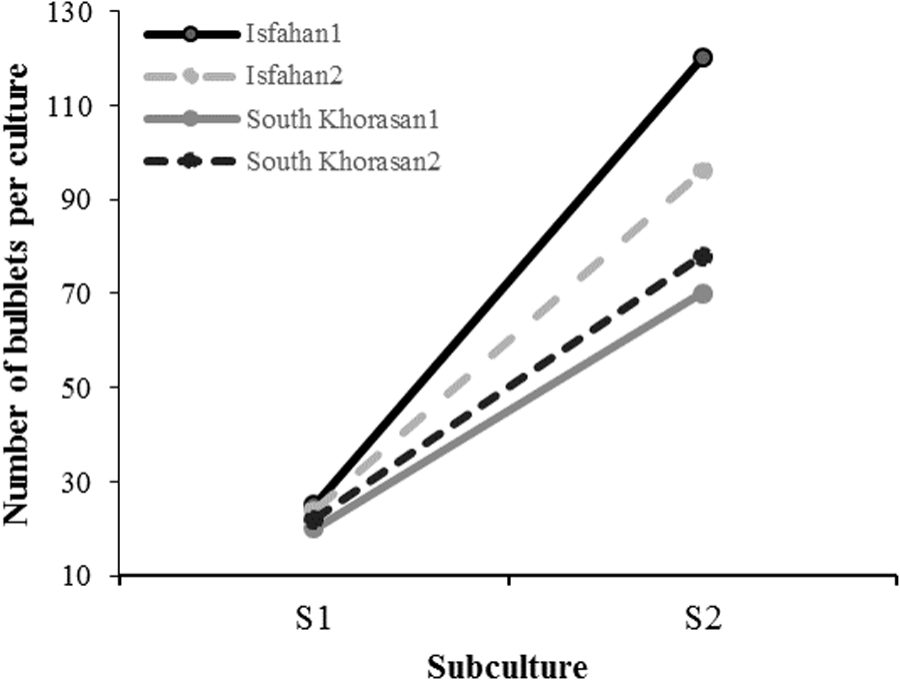
Mean number of bulblets per culture tube at conservation phase in Iranian garlic genotypes on media culture containing 0.1 mgL^-1^ NAA and 0.5 mgL^-1^ 2-iP. S1 and S2 showed first and second subculture, respectively.

To assess *in vitro* conservation capacity of Iranian garlic genotypes, shoot tips of all regenerated genotypes were subcultured on our optimized culture media containing 0.1 mgL^-1^ NAA and 0.5 mgL^-1^ 2-iP. Analysis of variance showed that effects of genotypes, subculturing stage and their interaction on number of bulblets per culture tube were significant (p < 0.01 probability) (Table 6). Surprisingly, the number of bulblets per culture tube increased dramatically from 20–25 in the first subculture to 70–120 in the second subculture, showing 4–5-fold increase in the frequency of regeneration. This is considered a remarkable achievement compared to regeneration frequencies reported in earlier studies [42]. The highest (125/culture tube) and lowest bulblets number (20/culture tube) were observed in genotypes Isfahan1 and South Khorasan1, respectively (Figs 5 and 6). An increased number of shoots has also been reported in repeated subcultures of garlic by Hailu et al [44], but in much lower frequency. Here, we report huge increase in bulblet formation, due to the repeated subculture of garlic.

**Table 6:** Analysis of variance comparing the effects of subculture and genotype on bulblets formation in conservation phase of *in vitro* garlic culture.

Source of variation	df	Sum of squares	Mean square	F
Genotype (G)	3	38.80	40.96	1.78
Subculture (S)	1	1197.39	1264.00	54.96
G * S	3	26.76	28.25	1.23
Experimental error	16	368.00	23.00	
Total	23	1631		

n.s means no statistical difference.

### Assessment of somaclonal variation in *in vitro* seedlings compared to maternal plants based on morphological quality traits

The result of analyzing the data from parental plants and their *in vitro* propagated progeny for four qualitative traits, evaluated based on the IPGRI international descriptor [33], indicated no visible differences between the parental plants and the *in vitro* propagated plants. In words, based on these qualitative morphological traits the four *in vitro* grown plants were true to type showing no somaclonal variation.

### Assessing somaclonal variation in regenerated garlic plantlets

*In vitro* propagated garlic seedlings were grown side by side with their *in vivo* grown parental clones under controlled conditions and were evaluated for their possible morphological variations using qualitative traits as described by IPGRI [33]. Based on this evaluation, no morphological variation was detected. So, all *in vitro* generated garlic plants examined here appeared to be true to type. This was then reassessed by RAPD markers using five most polymorphic primers examined in this study. RAPD markers are highly polymorphic and are commonly used for evaluating genetic variation including somaclonal variation in plants [25,45]. Four *in vitro* garlic clones were assessed against their original genotypes for detecting DNA polymorphism caused by somaclonal variation.

**Fig 7:**
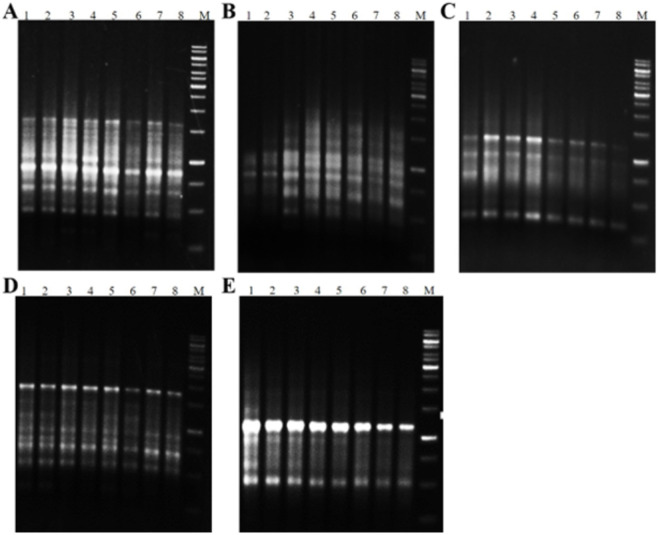
Electrophoretic banding patterns of four Iranian garlic genotypes with five RAPD primers ((A) OPA-02, (B) OPD-01, (C) OPJ-12, (D) K-15, (E) K-20). Lane 1: Isfahan1 mother plant, Lane 2: regenerated Isfahan1 *in vitro*, Lane 3: South Khorasan1 mother plant, Lane 4: regenerated South Khorasan1 *in vitro*, Lane 5: Isfahan2 mother plant, Lane 6: regenerated Isfahan2 *in vitro*, Lane 7: South Khorasan2 mother plant, Lane 8: regenerated South Khorasan2 *in vitro*, M: Molecular weight marker (1kb DNA ladder).

The electrophoretic patterns of PCR products which amplified by five RAPD primers were shown in Fig 7. The primers generated 2–9 bands. The same number of bands were amplified in both *in vitro* generated clones and their mother plants in all four genotypes. So, this indicated that all amplified DNA bands were monomorphic and somaclonal variation did not occur in micropropagated garlic clones despite the use of PGRs on MS medium containing 0.1 mg/L NAA and 0.5 mg/L 2-iP. Izquierdo-Oviedo et al [18], also used eight RAPD primers such as OPD-01 to assess possible somaclonal variation and observed monomorphic bands in both regenerated garlic plants in the MS medium with 0.1 mgL^-1^ NAA and 4 mgL^-1^ 2-iP and their original mother plant.

## Conclusion

This study revealed that solid MS medium with 3% (w/v) sucrose supplemented with 1.5 mgL^-1^ BA + 0.5 mgL^-1^ IBA or 0.5 mgL^-1^ 2-iP + 0.25 mgL^-1^ NAA were equally suitable culture media for establishing garlic *in vitro* shoot meristem culture. Moreover, similar MS medium supplemented with 0.1 mgL^-1^ NAA and 0.5 mgL^-1^ 2-iP appeared to be the most suitable medium for regeneration and conservation of Iranian garlic genotypes examined here. A multistep *in vitro* system for garlic micropropagation reported in this study has drastically increased the frequency of bulblets formation in garlics compared to results reported before. The study also showed that regenerated garlic plants were genetically stable as determined by RAPD markers. The results reported here pave the ground for developing a technically efficient and economically viable micropropagation system for conservation and breeding of garlic germplasm as well as the production of virus-free seed garlic.

## Supporting information

S1 Data Electrophoretic banding patterns of four Iranian garlic genotypes with five RAPD primers. (PDF)
