# Supplementary material for: Micropropagation and somaclonal variation in Iranian genotypes of garlic (Allium sativum L.)
Source: PLoS One. 2025 Sep 10;20(9):e0331782. doi: 10.1371/journal.pone.0331782 (PMC12422459; doi:10.1371/journal.pone.0331782)
Supplement: S1 Data — (PDF) [file pone.0331782.s001.pdf]

Fig 7. Electrophoretic banding patterns of four Iranian garlic genotypes with five RAPD primers

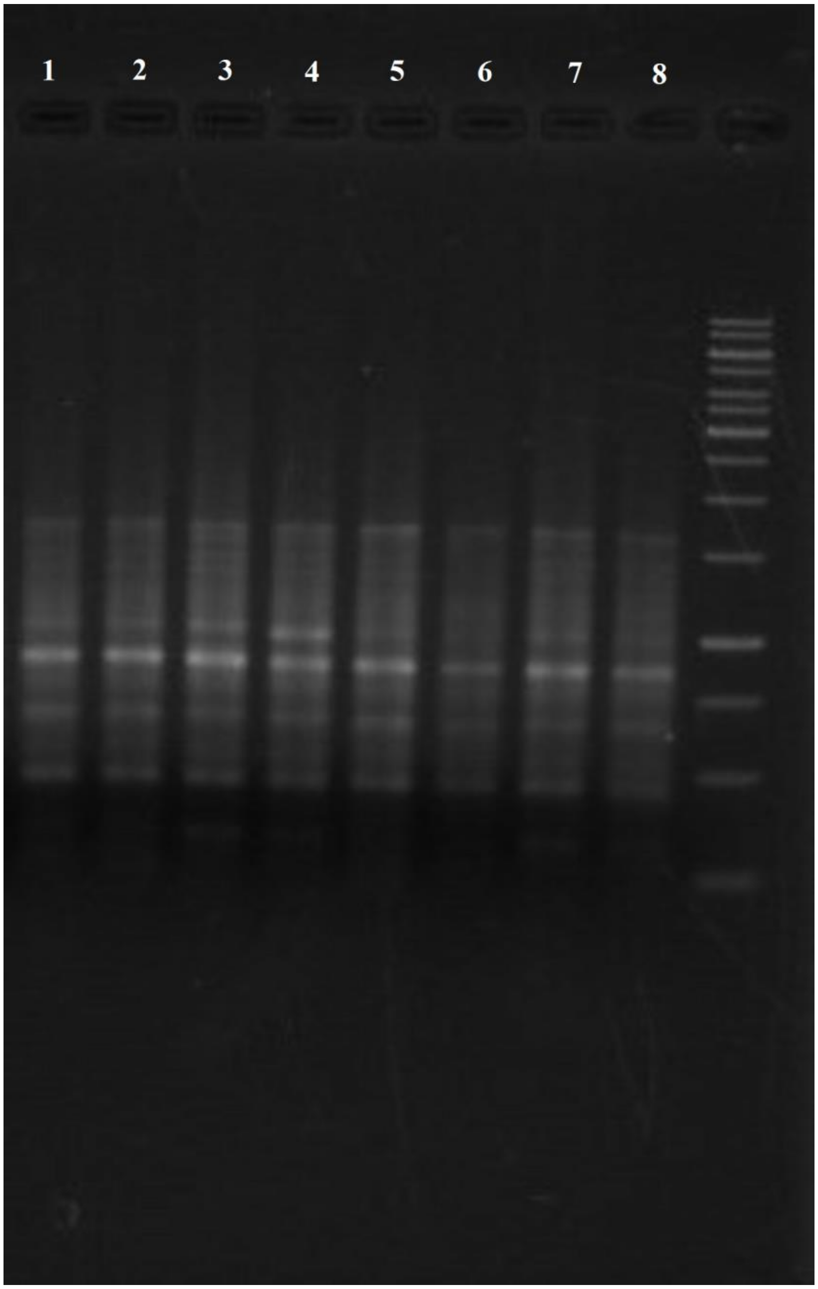

Fig 7A. Electrophoretic banding patterns of four Iranian garlic genotypes with OPA-02 primer.

Lane 1: Isfahan1 mother plant, Lane 2: regenerated Isfahan1 *in vitro*, Lane 3: South Khorasan1 mother plant, Lane 4: regenerated South Khorasan1 *in vitro*, Lane 5: Isfahan2 mother plant, Lane 6: regenerated Isfahan2 *in vitro*, Lane 7: South Khorasan2 mother plant, Lane 8: regenerated South Khorasan2 *in vitro*, and 1kb DNA ladder.

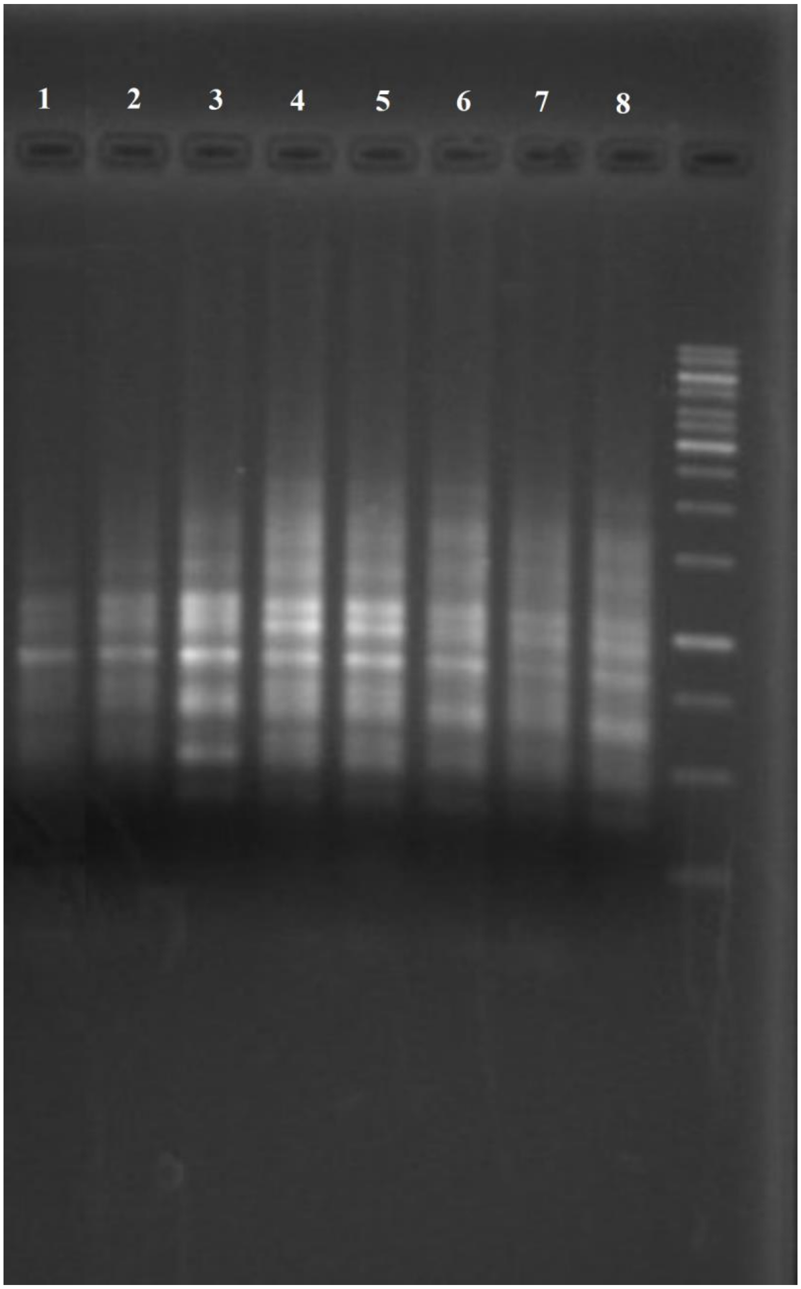

Fig 7 B. Electrophoretic banding patterns of four Iranian garlic genotypes with OPD-01 primer

Lane 1: Isfahan1 mother plant, Lane 2: regenerated Isfahan1 *in vitro*, Lane 3: South Khorasan1 mother plant, Lane 4: regenerated South Khorasan1 *in vitro*, Lane 5: Isfahan2 mother plant, Lane 6: regenerated Isfahan2 *in vitro*, Lane 7: South Khorasan2 mother plant, Lane 8: regenerated South Khorasan2 *in vitro*, and 1kb DNA ladder.

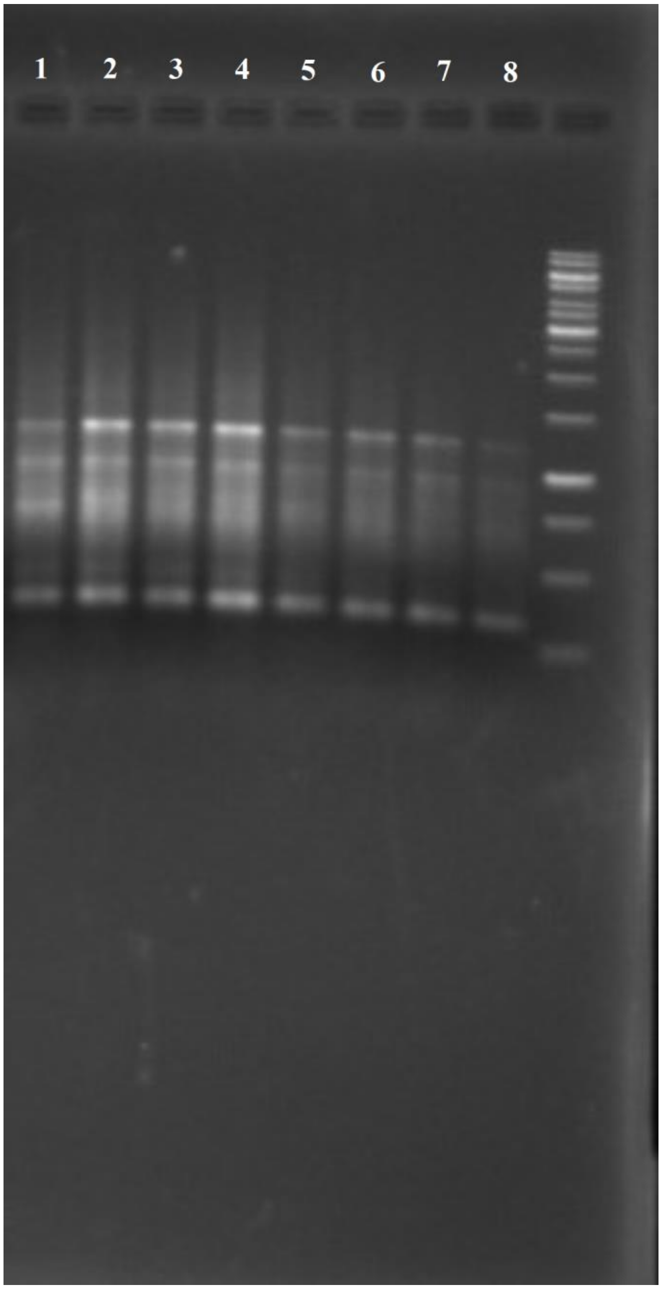

Fig 7 C. Electrophoretic banding patterns of four Iranian garlic genotypes with OPJ-12 primer

Lane 1: Isfahan1 mother plant, Lane 2: regenerated Isfahan1 *in vitro*, Lane 3: South Khorasan1 mother plant, Lane 4: regenerated South Khorasan1 *in vitro*, Lane 5: Isfahan2 mother plant, Lane 6: regenerated Isfahan2 *in vitro*, Lane 7: South Khorasan2 mother plant, Lane 8: regenerated South Khorasan2 *in vitro*, and 1kb DNA ladder.

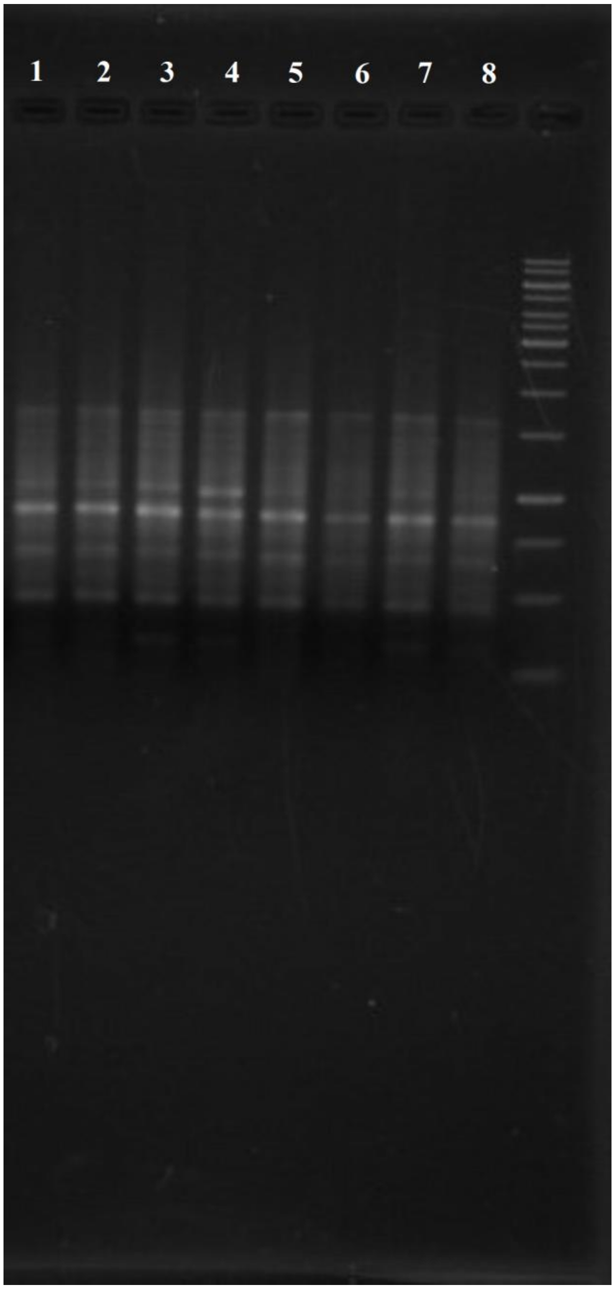

Fig 7 D. Electrophoretic banding patterns of four Iranian garlic genotypes with K-15 primer
Lane 1: Isfahan1 mother plant, Lane 2: regenerated Isfahan1 *in vitro*, Lane 3: South Khorasan1 mother plant, Lane 4: regenerated South Khorasan1 *in vitro*, Lane 5: Isfahan2 mother plant, Lane 6: regenerated Isfahan2 *in vitro*, Lane 7: South Khorasan2 mother plant, Lane 8: regenerated South Khorasan2 *in vitro*, and 1kb DNA ladder.

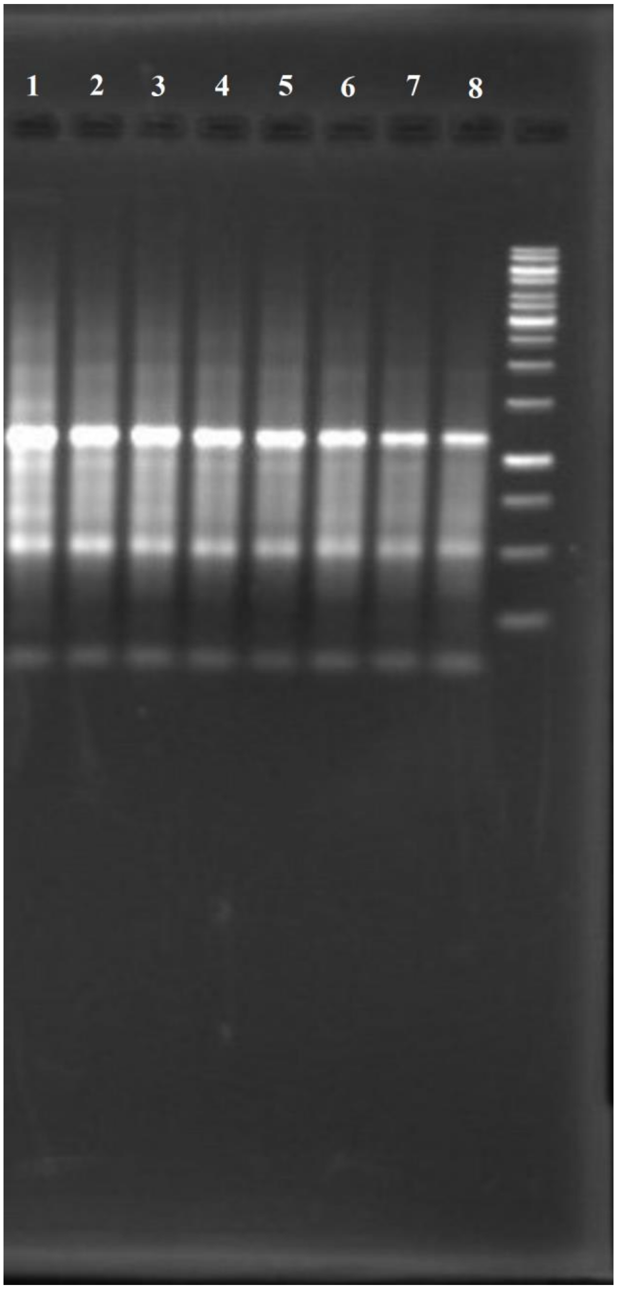

Fig 7 E. Electrophoretic banding patterns of four Iranian garlic genotypes with K-20 primer
Lane 1: Isfahan1 mother plant, Lane 2: regenerated Isfahan1 *in vitro*, Lane 3: South Khorasan1 mother plant, Lane 4: regenerated South Khorasan1 *in vitro*, Lane 5: Isfahan2 mother plant, Lane 6: regenerated Isfahan2 *in vitro*, Lane 7: South Khorasan2 mother plant, Lane 8: regenerated South Khorasan2 *in vitro*, and 1kb DNA ladder.
